# Serum Adiponectin and Leptin Concentrations in Relation to Body Fat Distribution, Hematological Indices and Lipid Profile in Humans

**DOI:** 10.3390/ijerph120911528

**Published:** 2015-09-14

**Authors:** Anna Lubkowska, Aleksandra Radecka, Iwona Bryczkowska, Iwona Rotter, Maria Laszczyńska, Wioleta Dudzińska

**Affiliations:** 1 Department of Functional Diagnostics and Physical Medicine, Faculty of Health Sciences, Pomeranian Medical University in Szczecin; ul. Żołnierska 54, 71–210 Szczecin, Poland; E-Mails: aleksandradobek@gamil.com (A.R.); ibryczkowska@gmail.com (I.B.); 2 Department of Medical Rehabilitation, Faculty of Health Sciences, Pomeranian Medical University in Szczecin; ul. Żołnierska 54, 71–210 Szczecin, Poland; E-Mail: iwrot@wp.pl; 3 Department of Histology and Developmental Biology, Faculty of Health Sciences, Pomeranian Medical University in Szczecin; ul. Żołnierska 48, 71–210 Szczecin, Poland; E-Mail: laszcz@pum.edu.pl; 4 Department of Physiology, Faculty of Biology, Szczecin University, ul. Felczaka 3c, 71–412 Szczecin, Poland; E-Mail: wiola@univ.szczecin.pl

**Keywords:** adiponectin, leptin, ADPN/LEP ratio, lipid profile, body mass components

## Abstract

The purpose of the study was to evaluate the relationship between serum adiponectin and leptin concentrations and body composition, hematological indices and lipid profile parameters in adults. The study involved 95 volunteers (BMI from 23.3 to 53 kg/m^2^). Anthropometric parameters were measured: body weight and height, waist and hip circumference, waist-to-hip ratio, body fat mass (BMF), subcutaneous and visceral fat mass (SFM, VFM), lean body mass (LBM), skeletal muscle mass (SMM). In serum we determined adiponectin and leptin concentrations, extracellular hemoglobin, total bilirubin, as well as lipid metabolism (TCh, HDL-Ch, LDL-Ch, TG). Mean adipokine levels were significantly higher in women (*p* ≤ 0.01), adiponectin significantly negatively correlated with body height and weight, systolic blood pressure and absolute LBM and SMM values. The same relation was observed for erythroid system indicators and lipid indicators. A positive correlation was exceptionally found between adiponectin and HDL-Ch. LEP negatively correlated with some percentage rates (%LBM, %SMM). Only in women, we observed a positive correlation between LEP and body weight, BMI and WHR. Studies on ADPN and the ADPN/LEP ratio as a valuable complementary diagnostic element in the prediction and prevention of cardiovascular diseases need to be continued.

## 1. Introduction

Cardiovascular disease (CVD), like other chronic diseases, is the result of complex interactions between genetic and environmental factors over extended periods of time. CVD risk factors include metabolic (e.g., total cholesterol, HDL cholesterol, fasting blood glucose, insulin resistance), biological (e.g., blood pressure), as well as lifestyle factors (physical inactivity, diet, tobacco smoking) [1]. Yet these classical risk factors only partly explain high cardiovascular risk and, in order to enhance the understanding of CVD (and its treatment, too), research focusing on traditional as well as novel risk factors is still necessary to determine the most effective means of reducing unhealthy metabolic profiles.

The discovery of the endocrine function of adipose tissue has boosted the interest in its role in CVD and resulted in the reevaluation of many preconceived notions about this tissue [2]. Adipocytes are now known to have numerous receptors responsible for their sensitivity to humoral factors, allowing interactions between adipose tissue and the endocrine, cardiovascular, immune and nervous systems [3]. The significance of the endocrine function of adipose tissue is particularly evident in diseases accompanying abdominal obesity, diseases associated with dyslipidemia, elevated blood pressure, activation of prothrombotic and inflammatory processes, insulin resistance, atherosclerosis and impaired glucose tolerance, and therefore with type 2 diabetes (T2DM) and development of metabolic syndrome [3,4,5]. In addition, significant differences have been shown in recent years between subcutaneous and visceral fat, both in terms of the endocrine function and the released products [6,7].

The clinical significance of abdominal obesity is well known and is associated with adverse changes in lipid indicators and its relationship to increased risk of coronary heart disease, hypertension, metabolic syndrome and strokes [8]. Adipocytes secrete a variety of biologically active molecules which may influence the function and the structural integrity of the cardiovascular system, and may be involved in cardiovascular risk. For example, it is postulated that adiponectin (ADPN) and leptin (LEP) levels, as well the ADPN/LEP ratio, are associated with BMI and body fat, although literature data are often ambiguous. Nonetheless, the observed trends indicate that these adipokines may be used as adjunctive markers of metabolic disorders being associated with cardiovascular pathologies.

ADPN and LEP have a common tissue origin and protein structure. Their other common feature is their distribution by adipocytes, and lesser known distribution by skeletal muscles and myocardium [9,10,11]. However, their activities and roles are quite different. ADPN increases insulin sensitivity and fatty acid oxidation, reduces glucose synthesis and enhances glucose uptake by the liver and other tissues [12,13]. LEP is mainly responsible for the regulation of food intake [9]; its higher serum levels inhibit hunger, increase energy expenditure by raising body temperature, while increasing fatty acid oxidation in the liver and skeletal muscle [14]. LEP has also been shown to be involved in immunological processes, hematopoiesis and probably in pathological processes, autoimmune diseases [15,16], angiogenesis, hemostasis and wound healing [17,18].

It is possible that ADPN and LEP may be the risk markers for fat-induced dyslipidemia or insulin resistance, with a risk for type 2 diabetes and cardiovascular disease. However, despite a vast body of research, the role of LEP and ADPN in the pathogenesis of obesity and cardiovascular diseases still raises a lot of controversies, for example due to discrepancies between clinical and animal research findings [19,20,21].

A recent study has shown a negative correlation of serum adiponectin level and a positive correlation of serum leptin level with visceral fat area in adults and these correlations are more significant compared to BMI ones. Leptin and adiponectin, among various adipocyte-derived cytokines, are thought to be involved in the regulation of metabolic homeostasis. Additionally, the evaluation of the leptin: adiponectin ratio has been suggested as a useful parameter for assessing insulin resistance, and is more effective as an insulin resistance parameter than adiponectin or leptin alone. In this study we performed a multifaceted analysis of the relationships between ADPN, LEP, and the ADPN/LEP ratio, and the selected markers of metabolic syndrome, including a broad blood biochemical profile and hematological parameters, being accompanied by a detailed analysis of body composition in people of varying body weight and allowing for differences between the sexes. In estimating the body composition, we included the percentage of fat components (including subcutaneous and visceral fat) or non-fat components, and their segmental distribution.

## 2. Methods

### 2.1. Participants

The study involved 95 volunteers, non-diabetic individuals, comprising 55 women aged 38 (from 23 to 54) years (BMI = 31.6 kg/m^2^; from 23.3 to 53 kg/m^2^) and 40 men aged 32 (from 24 to 58) years (BMI = 31.9 kg/m^2^; from 24.6 to 41.2 kg/m^2^). An inclusion criterion for participation in the study was: not taking medications on a permanent basis or contraceptives in the case of women. The study was approved by the local Ethics Committee (Pomeranian Medical University; Ref. KB-0012/54/10).

### 2.2. Measures

#### 2.2.1. Anthropometric Measurements and Bioelectrical Impedance Analysis (BIA)

For each of the subjects we performed basic anthropometric measurements: body weight, body height, waist and hip circumference, and waist-hip ratio (WHR). A bioelectrical impedance analysis (using a Medical X-ScanPlus II, Jawon, Kyungsan City, Korea) was used to estimate body composition parameters: body fat mass (BFM), subcutaneous fat mass (SFM), visceral fat mass (VFM), lean body mass (LBM), skeletal muscle mass (SMM) and the percentage content of these components. In addition, the following parameters were determined in the trunk: soft lean mass (SLM_T_), body fat mass (MBF_T_) and subcutaneous fat mass (SFM_T_), and in the lower limb region: body fat mass for the right and left legs (BFM_RL_, MBF_LL_). We also determined the proportion of the trunk and limb components in the total body mass of subjects under study. Additionally, we calculated the ratio of subcutaneous to visceral fat in the whole body and trunk (SFM:VFM, SFM_T_:VFM_T_), and the ratio of body fat mass to lean body mass (BFM:LBM). The measurement was carried out using eight surface electrodes connected to the analyzer, using electric current (frequency 1 kHz to 1000 kHz at 180μA). Electrodes in this method are placed as a tetrapolar system with the opposite arrangement, four electrodes built into the hand brackets on the apparatus (two electrodes per hand), and four on a measuring platform (two per foot). The study was conducted in accordance with the standards for the measurement procedure, at the same time of the day for all subjects, in the morning on an empty stomach. According to literature, BIA techniques were found to be particularly precise when compared with DEXA and appear to be a robust tool for measuring and monitoring total body fat and lean body mass in clinical and healthy volunteer studies [22,23]

Resting blood pressure (BP) was measured three times in the seated position (M2 Basic, OMRON HEALTHCARE Co., Ltd, Kyoto, Japan). The average of three readings was used as the representative examination value. The measurement was performed under controlled conditions in a quiet room.

#### 2.2.2. Biochemical Parameters of Venous Blood

Venous blood was collected from each of the volunteers, after overnight fasting, between 7.00 a.m. and 8.30 a.m. in the morning, after a 10 min rest in a sitting position, from the antecubital vein using Vacutainer tubes (Sarstedt, Germany), separately into two tubes: one to determine blood counts (1.2 mL anticoagulated with 1 g/L K2 EDTA) and the other for biochemical analysis of serum (7 mL).

Serum levels of adiponectin and leptin were measured by immuno-enzymatic assays using commercially available ELISA kits from R&D Systems (Abingdon, UK), according to the manufacturer’s instructions. Adiponectin assay sensitivity was 0.246 ng/mL, while intra and inter-assay coefficients of variation (CV) were 2.5%–4.7% and 5.8%–6.9%, respectively. Leptin assay sensitivity was 7.8 pg/mL, and intra and inter-assay CVs were 3.3%–3.2% and 4.2%–3.5%, respectively.

A spectrophotometric method was used to determine extracellular hemoglobin, total bilirubin, total protein, albumin, total cholesterol, HDL cholesterol and triglycerides, while the concentration of LDL cholesterol fraction was determined by a direct method. After determinations, the obtained lipid profile was supplemented by calculation of TCh:HDL, LDL:HDL and TG:TCh ratios.

#### 2.2.3. Statistical Analysis

Statistical analysis of the results was performed using STATISTICA software (version 10 PL). In addition to descriptive statistics (median, minimum, maximum), we tested the normality of the distribution of analyzed parameters using the Shapiro-Wilk test. Since the distribution of the majority of measured values deviated from a normal distribution, the comparison between men and women was performed with the non-parametric Mann-Whitney U-test, taking into account the Bonferroni correction. To assess the strength and direction of the relationships between parameters and ADPN and LEP concentrations and the ADPN/LEP ratio, we calculated the Spearman rank correlation coefficients. The significance level was assumed at *p* < 0.05. In order to check the homogeneity of the examined group of women and men, we calculated the coefficients of variation (CV) for all analyzed parameters.

## 3. Results

### 3.1. Anthropomorphic Parameters in the Study Group

The study group of women differed significantly from that of men in terms of height and weight. Similarly, sexual differentiation was observed in blood pressure ranges which were significantly higher in men (mean value of 140/80 mmHg; SBP *p* < 0.001, DBP *p* < 0.05). Other anthropometric parameters, including age, BMI and WHR, showed no differences between sexes. Highly statistically significant (*p* < 0.001) sex-related differences were demonstrated in absolute non-fat components (LBM, SMM, SLM_T_), while no differences were demonstrated for the absolute levels of all analyzed indicators related to body fatness (MBF, MBFT, VFM, SFM). With respect to the percentages of the determined components, significant differences between sexes were shown again for all lean indicators (%LBM, %SMM, %SLM_T_), as well as for most indicators related to fat (%BFM, %SFM, %BFM_T_, %SFM_T_), except—interestingly—the percentage of visceral adipose tissue (%VFM).

The comparison of the ratio of subcutaneous to visceral fat mass for the whole body (SFM:VFM) and only for the trunk (SFM_T_:VFM_T_) showed significant sex-related differences, indicating a greater percentage rate of subcutaneous fat in women than men, both for the whole body and the trunk. Similar differences and a greater fat content in women were found in the ratio of body fat to lean body mass (MBF:LBM). Similarly, the comparison of adiposity in the lower limb region (%MBF_RL_, %MBF_LL_) revealed significant differences between the sexes and a greater fat content in women; interestingly, the absolute fat mass in the analyzed regions (MBF_RL_, MBF_LL_) did not differ significantly between men and women. These results are summarized in Table 1.

**Table 1:** Anthropomorphic parameters in the study group, by sex.

Parameter	Female and Male n = 95	Female n = 55	Male n = 40
	Median (Range)	Median (Range)	Median (Range)
**Age [years]**	37 (23–58)	38 (23–54)	32 (24–58)
**Height [cm]**	170 (153–193)	168 (153–178)	178.0 ^***F^ (158–193)
**Weight [kg]**	95.25 (61.0–139)	81.90 (61–139)	97.60 ^*F^ (66.9–135.1)
**BMI [kg/m^2^]**	30.35 (23.3–53)	30.20 (23.3–53)	31.9 (24.6–41.2)
**WHR**	0.85 (0.82–1.09)	0.89 (0.82–1.03)	0.91 (0.84–1.09)
**SBP[mm Hg]**	130 (105–160)	125 (105–140)	140.0 ^***F^ (125–160)
**DBP[mm Hg]**	80 (70–100)	75 (70–85)	80.0 ^*F^ (70–100)
**LBM [kg]** **[%]**	58.30 (40.6–96.5) 64.82 (51.58–77.36)	51.30 (40.6–71.7) 61.17 (51.58–68.87)	70.9 ^***F^ (43.20–96.5) 68.78 ^***F^ (62.80–77.36)
**SMM [kg]** **[%]**	25.75 (13.3–50.0) 27.487 (19.11–41.51)	23.20 (13.9–30.9) 25.10 (19.11–37.2)	33.40 ^***F^ (13.3–50.0) 30.70 ^**F^ (19.88–41.51)
**VFM [kg]** **[%]**	4.45 (2.4–12.4) 5.26 (3.18–8.92)	4.60 (2.4–12.4) 5.46 (3.52–8.92)	4.30 (3.1–9.6) 4.63 (3.18–7.19)
**SFM [kg]** **[%]**	25.85 (18.0–54.9) 30.67 (19.47–39.49)	27.20 (18.0–54.9) 33.21 (27.61–39.5)	22.3 (18.5–40.1) 25.84 ^***F^ (19.47–30.79)
**SFM:VFM**	5.91 (3.91–7.83)	6.15 (4.43–7.83)	5.44 ^**F^ (3.99–6.65)
**MBF:LBM**	0.54 (0.29–0.94)	0.63 (0.45–0.94)	0.45 ^***F^ (0.29–0.59)
**SLM_T_ [kg]** **[%]**	26.26 (18.94–42.73) 29.43 (22.12–35.66)	23.37 (18.94–31.26) 27.85 (22.12–31.98)	31.68 ^***F^ (20.30–42.73) 31.63 ^***F^ (27.86–35.66)
**MBF_T_ [kg]** **[%]**	15.74 (10.49–34.52) 18.09 (11.62–24.83)	16.31 (10.49–34.52) 19.915 (16.04–24.83)	13.67 (11.25–25.56) 16.05 ^***K^ (11.62–19.13)
**SFM_T_ [kg]** **[%]**	11.08 (7.85–22.12) 13.64 (8.44–15.91)	11.71 (8.09–22.12) 14.37 (12.51–15.91)	9.57 (7.85–15.96) 10.61 ^***F^ (8.44–13.57)
**SFM_T_:VFM_T_**	2.55 (1.57–3.55)	2.68 (1.78–3.55)	2.31^**F^ (1.57–2.93)
**MBF_RL_ [kg]** **[%]**	5.57 (3.70-11.94) 6.36 (4.10–8.59)	5.73 (3.70–11.94) 6.99 (5.64–8.59)	4.81 ^**^M^LL MBF^ (3.99–8.99) 5.64 ^***F^, ^**^ M^LL MBF^(4.1–6.73)
**MBF_LL_ [kg]** **[%]**	5.58 (3.70–11.93) 6.42 (4.08–8.58)	5.68 (3.70–11.93) 6.97 (5.64–8.58)	4.86 (3.98–9.04) 5.64 ^***F^ (4.08–6.77)

**BMI**: Body Mass Index; **WHR**: Waist:Hip Ratio; **LBM**: Lean Body Mass; **MBF**: Body Fat Mass; **SMM**: Skeletal Muscle Mass; **VFM**: Visceral Fat Mass; **SFM**: Subcutaneous Fat Mass; parameters were determined in the trunk: **SLM_T_**: Soft Lean Mass; **MBF_T_**: Body Fat Mass; **SFM_T_**: Subcutaneous Fat Mass; **MBF_RL_**, **MBF_/LL_**: Body Fat Mass for the Right and Left Legs; *F-p<0.05 *vs*. Female; **F-p<0.01 *vs.* Female; ***F-p<0.001 *vs.* Female; **M^LLMBF^-p<0.01 *vs*. Meale Body Fat Mass for the Left Leg.

### 3.2. Clinical and Biochemical Characteristics of the Study Population

The main clinical and biochemical characteristics of the study population are summarized in Table 2. The analysis of serum adipokine concentrations showed significantly higher mean levels in women (*p* < 0.01 for ADPN, *p* < 0.001 for LEP). Considering the values of hematological indices, we observed expected differences between men and women in relation to RBC (*p* < 0.01), HGB (*p* < 0.001) and HCT (*p* < 0.001). The values of other hematological parameters were comparable in both sexes. In addition, differences between the sexes were shown in extracellular hemoglobin (*p* < 0.01) and total bilirubin (*p* < 0.05), the concentrations of which were higher in men. Albumin and total protein concentrations did not differ significantly between the sexes. Analysis of the lipid profiles showed significant differences in HDL cholesterol (*p* < 0.05) and TG (*p* < 0.01) which were higher in women than in men. In addition, significant sex-related differences were shown for the TG:TCh ratio, with higher values for men (*p* < 0.05). Other parameters of the lipid profile (LDL, TCh:HDL, LDL:HDL) were similar for women and men. The results are shown in Table 2.

**Table 2:** Clinical and biochemical characteristics of the study group.

Parameter	Female and Male n = 95	Female n = 55	Male n = 40
	Median (Range)	Median (Range)	Median (Range)
**Adiponectin[ng/mL]**	41.37 (6.01–145.40)	59.40 (16.37–145.40)	34.98 ^**F^ (6.01–85.53)
**Leptin [pg/mL]**	221.85 (45.68–894.50)	255.30 (98.83–894.50)	102.50 ^***F^ (45.68–430.70)
**ADPN/LEP ratio**	0.2 (0.03–1.28)	0.2 (0.03–0.86)	0.2 (0.11–1.27)
**RBC [10^12^/L]**	4.92 (3.85–5.89)	4.76 (3.85–5.46)	5.20 ^**F^ (4.49–5.89)
**HGB [mmol/L]**	7.65 (5.00–9.00)	7.50 (5.00–8.00)	8.40 ^***F^ (7.20–9.00)
**HCT [L/L]**	0.41 (0.29–0.51)	0.40 (0.29–0.43)	0.45 ^***F^ (0.39–0.51)
**MCV [fL]**	85.00 (70.00–93.00)	84.00 (70.00–92.00)	86.00 (82.00–93.00)
**MCH [fmol]**	1.57 (1.19–1.77)	1.55 (1.19–1.73)	1.59 (1.49–1.77)
**MCHC [mmol/L]**	18.60 (17.10–19.60)	18.60 (17.10–19.10)	18.70 (17.20–19.60)
**RDW [%]**	12.70 (10.90–16.30)	12.80 (10.90–16.30)	12.60 (11.50–13.50)
**WBC [10^9^/L]**	7.25 (5.00–10.20)	7.80 (5.00–10.20)	6.70 (5.70–8.50)
**LYM [10^9^/L]**	2.60 (1.80–4.20)	2.60 (1.80–4.20)	2.60 (1.90–3.20)
**MON [10^9^/L]**	0.50 (0.30–0.80)	0.50 (0.30–0.80)	0.50 (0.40–0.80)
**GRA [10^9^/L]**	4.15 (2.50–6.20)	4.30 (2.50–6.20)	3.800 (2.70–5.30)
**Extracellular hemoglobin [g/dL]**	16.19 (8.76–110.48)	13.33 (8.76–110.48)	17.14 ^**F^ (14.10–36.19)
**Total bilirubin [mg/dL]**	2.36 (1.24–7.20)	2.25 (1.35–4.16)	3.60 ^*F^ (1.24–7.20)
**Total protein [g/L]**	52.88 (21.24–67.35)	53.333 (21.24–67.35)	51.98 (33.45–57.85)
**Albumin [g/L]**	38.87 (19.72–59.95)	36.53 (19.72–59.95)	40.53 (26.15–55.89)
**Cholesterol [mg/dL]**	206.50 (123.00–314.00)	205.00 (142.00–287.00)	222.00 (123.00–314.00)
**HDL [mg/dL]**	28.32 (7.08–41.59)	30.09 (16.37–41.59)	25.66 ^*F^ (7.08–34.07)
**LDL [mg/dL]**	147.43 (39.58–238.80)	148.74 (102.16–238.80)	133.97 (39.58–228.05)
**TG [mg/dL]**	132.61 (37.68–372.46)	118.84 (37.68–297.10)	171.01 ^**F^ (89.86–372.46)
**TCh:HDL**	7.12 (4.40–34.32)	6.66 (4.40–14.90)	8.16 (5.17–34.32)
**LDL:HDL**	4.99 (2.24–26.61)	4.96 (2.85–11.37)	5.13 (2.24–26.61)
**TG:TCh**	0.64 (0.24–2.87)	0.58 (0.24–1.21)	0.96 ^*F^ (0.41–2.87)

**RBC**: Red Blood Cells; **HGB**: Hemoglobin; **HCT**: Hematocrit; **MCV**: Mean Corpuscular Volume; **MCH**: Mean Corpuscular Hemoglobin; **MCHC**: Mean Corpuscular Hemoglobin Concentration; **RDW**: Red blood cell Distribution Width; **WBC**: White Blood Cells; **LYM**: Lymphocytes; **MON**: Monocytes; **GRA**: Granulocytes; **PLT**: Platelets; *F-p<0.05 *vs.* Female; *F-p<0.01 *vs.* Female; ***F-p<0.001 *vs*. Female.

### 3.3. Relationships between the Analyzed Anthropomorphic Parameters and Serum Adiponectin and Leptin Concentrations

The search for relationships between the analyzed anthropomorphic parameters was conducted for the entire study group (95 persons) and separately for women and men. Analysis of the results showed no relationship between fasting ADPN and LEP concentrations in serum, either in the entire population, or in women and men groups (Table 3). In the entire group of participants we found a significant negative correlation between ADPN and body height, body weight, systolic blood pressure and the absolute values of all analyzed lean body components: LBM, SMM and SLM_T_. The relationships were partly repeated in the group of men, as shown by a negative correlation between ADPN and body height and lean body components (LBM, SLM_T_). However, the group of women did not show such trends. Interestingly, a positive correlation between the LEP level and body weight, BMI and WHR was found in the group of women only. In the entire group of subjects, LEP negatively correlated with the percentage of all analyzed lean body components and, taking into account only the absolute values, LEP negatively correlated with muscle mass only. Interestingly, there was a positive correlation in women between lean body mass (LBM) and soft lean mass (SLM_T_) and LEP, which was not demonstrated either in the entire study group or in men.

A positive correlation was found between LEP and all fat mass components expressed in absolute values (MBF, VFM, SFM, MBF_T_, SFM_T_, MBF_RL_, MBF_LL_) and for most percentage rates (%VFM, %SFM, %MBF_T_, SFM_T_, %MBF_RL_, %MBF_LL_). The trend being shown for the absolute values of analyzed fat indicators was largely repeated in separately analyzed groups of women and men, except visceral fat mass (VFM) in men. Similarly, with regard to the percentage of fat components analyzed separately for women and men, we found a positive correlation between LEP and all above body fat parameters, and additionally the percentage rate of body fat mass (%MBF), which was not observed for the entire study group.

**Table 3:** Significant correlations (*p* < 0.05) between serum adiponectin (ADPN) and leptin (LEP) and selected parts of the body, including division into sex groups.

Parameter	Female and Male n = 95			Female n = 55			Male n = 40		
	ADPN [ng/mL]	LEP [pg/mL]	ADPN/LEP Ratio	ADPN [ng/mL]	LEP [pg/mL]	ADPN/LEP Ratio	ADPN [ng/mL]	LEP [pg/mL]	ADPN/LEP Ratio
**Height [cm]**	–0.33	-	-	-	-	-	–0.59	-	-
**Weight [kg]**	–0.42	-	–0.33	-	0.59	-	-	-	–0.52
**BMI [kg/m^2^]**	-	-	–0.42	-	0.59	–0.52	-	-	-
**WHR**	-	-	-	-	0.57	–0.42	-	-	-
**SBP [mmHg]**	–0.49	-	-	-	-	-	-	-	-
**LBM [kg]/[%]**	–0.50/-	-/–0.81	-/-	-/-	0.55/–0.64	-/-	–0.62/-	-/–0.71	–0.54/-
**MBF [kg]/[%]**	-/-	0.54/-	–0.47/-	-/-	0.62/0.65	–0.42/-	-/-	0.54/0.71	-/-
**SMM [kg]/[%]**	–0.49/-	–0.37/–0.62	-/-	-/-	-/-	-/-	-/-	-/–0.59	-/-
**VFM [kg]/[%]**	-	0.34/0.56	–0.41/-	-/-	0.61/0.63	–0.47/-	-/-	-/0.55	-/-
**SFM [kg]/[%]**	-/-	0.58/0.81	–0.49/-	-/-	0.62/0.64	–0.42/-	-/-	0.60/0.69	–0.55/-
**SFM:VFM**	-	-	-	-	–0.57	-	-	-	-
**MBF:LBM**	-	0.8	-	-	0.64	-	-	0.71	-
**SLM_T_ [kg]/[%]**	–0.48/-	-/–0.77	-/0.43	-/-	0.54/–0.63	-/0.49	–0.59/-	-/–0.69	–0.52/-
**MBF_T_[kg]/[%]**	-/-	0.54/0.80	–0.47/–0.42	-/-	0.62/0.64	–0.42/–0.48	-/-	0.53/0.71	-/–0.53
**SFM_T_ [kg]/[%]**	-/-	0.61/0.81	–0.51/–0.39	-/-	0.63/0.66	–0.42/–0.49	-/-	0.65/0.73	–0.63/–0.58
**SFM_T_:VFM_T_**	-	-	-	-	–0.58	0.40	-	-	-
**MBF_RL_ [kg]/[%]**	-/-	0.53/0.8	-/-	-/-	0.60/0.64	-/-	-/-	0.56/0.68	-/-
**MBF_LL_ [kg]/[%]**	-/-	0.53/0.81	-/-	-/-	0.62/0.68	-/-	-/-	0.55/0.68	-/-

**BMI**: Body Mass Index; **WHR**: Waist:Hip Ratio; **LBM**: Lean Body Mass; **MBF**: Mass of Body Fat; **SLM**: Soft Lean Mass; **SMM**: Skeletal Muscle Mass; **VFM**: Visceral Fat Mass; **SFM**: Subcutaneous Fat Mass; _RL_-Right Leg; _LL_-Left Leg, _T_-Trunk.

In addition, in order to more fully determine the proportion of individual fat and lean tissue in the body, we analyzed the relationship between adipokines (ADPN and LEP) and the ratio of subcutaneous to visceral fat being assessed for the whole body (SFM:VFM) and the trunk (SFM_T_:VFM_T_) and the ratio of body fat mass to lean body mass (BFM:LBM). The analysis revealed a negative correlation between LEP and the SFM:VFM and SFM_T_:VFM_T_ ratios and a positive relationship between LEP and the MBF:LBM ratio in women only. These results are summarized in Table 3.

The ADPN/LEP ratio showed a negative correlation with body weight and BMI in the entire group of participants and, additionally, a negative correlation with WHR in women. In the group of men, this ratio negatively correlated with body mass only. Similarly, a negative correlation was demonstrated between the ADPN/LEP ratio and the absolute values of all analyzed body fat components (MBF, VFM, SFM, MBF_T_, SFM_T_) in the entire group and in women. In men, the ADPN/LEP ratio negatively correlated only with subcutaneous fat mass in the whole body and in the trunk (SFM, SFM_T_). In relation to the percentage rates of fat components, the entire group of participants and the sexes demonstrated a negative correlation between the ADPN/LEP ratio and subcutaneous fat mass in the trunk (%MBF_T_, %SFM_T_). It is interesting that, despite the strong relationship between the ADPN/LEP ratio and body fat components, a significant positive correlation was also shown with the percentage of soft lean mass in the trunk region (%SLM_T_) in the entire study group as well as in women. In men, this trend was not confirmed; on the contrary, a negative correlation was observed between the ADPN/LEP ratio and the absolute value of SLM_T_ and lean body mass (LBM) (Table 3).

**Figure 1:**
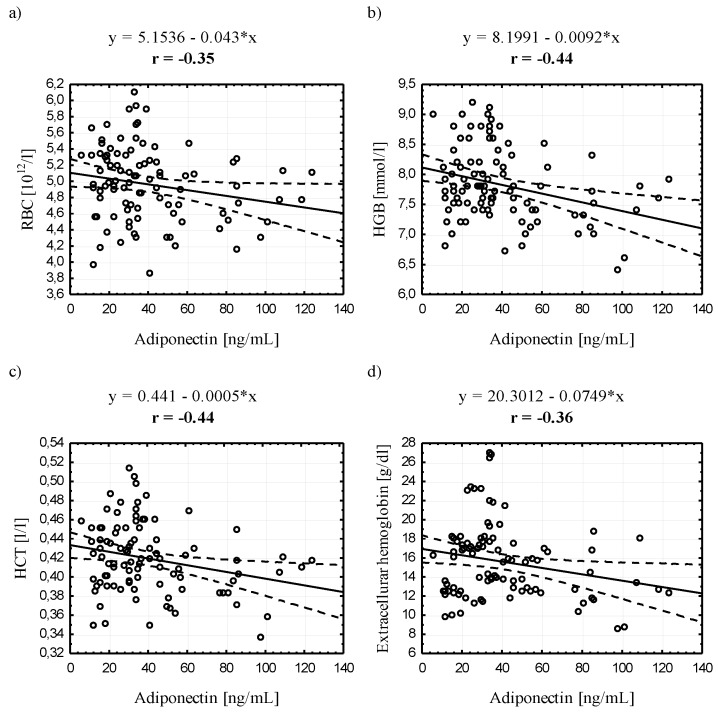
Scatter plots for the correlation (*p* < 0.05) between adiponectin and selected erythroid indicators in the study group.

### 3.4. Relationships between Serum Adiponectin and Leptin Concentrations and Selected Blood Biochemical Indicators

**Figure 2:**
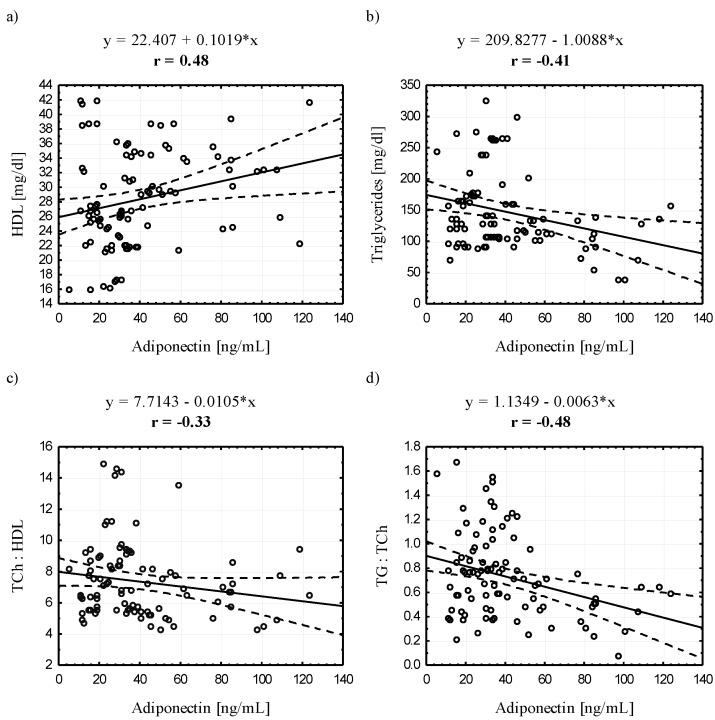
Scatter plots for the correlation (*p* < 0.05) between adiponectin and selected blood lipid profile indicators.

**Figure 3:**
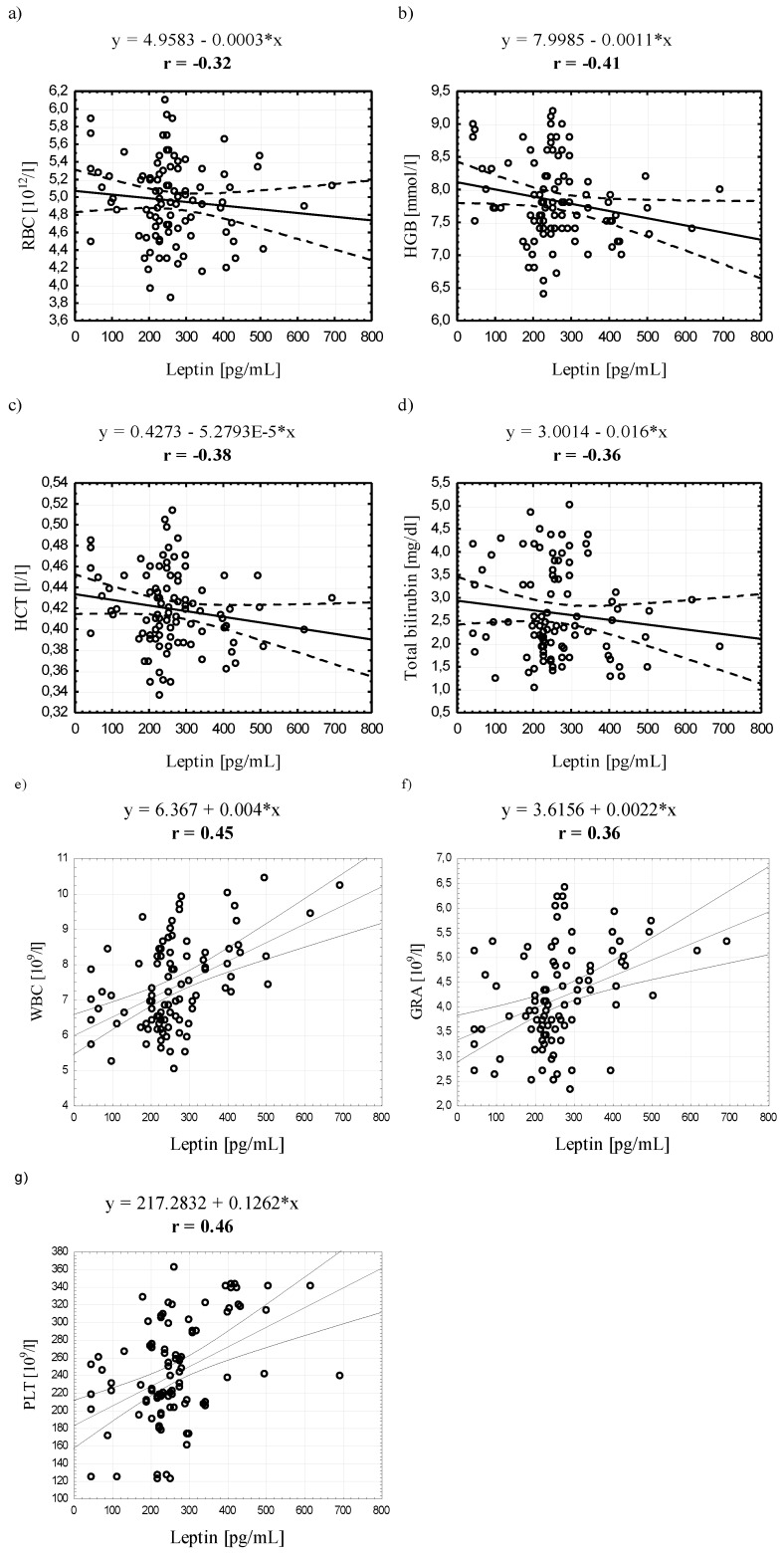
Scatter plots for the correlation (*p* < 0.05) between leptin and selected red and white blood cell indicators in the entire study group.

When analyzing the entire study group, we found a negative correlation between ADPN and erythroid indicators: RBC, HGB, HCT, and extracellular hemoglobin and lipid indicators: TG, TCh:HDL and TG:TCh, and a positive correlation with HDL. Similarly, LEP negatively correlated with these hematological indices and, additionally, with total bilirubin. Importantly, a positive correlation was found between LEP and white blood cell parameters: WBC, GRA and PLT (Figure 1, Figure 2 and Figure 3).

**Figure 4:**
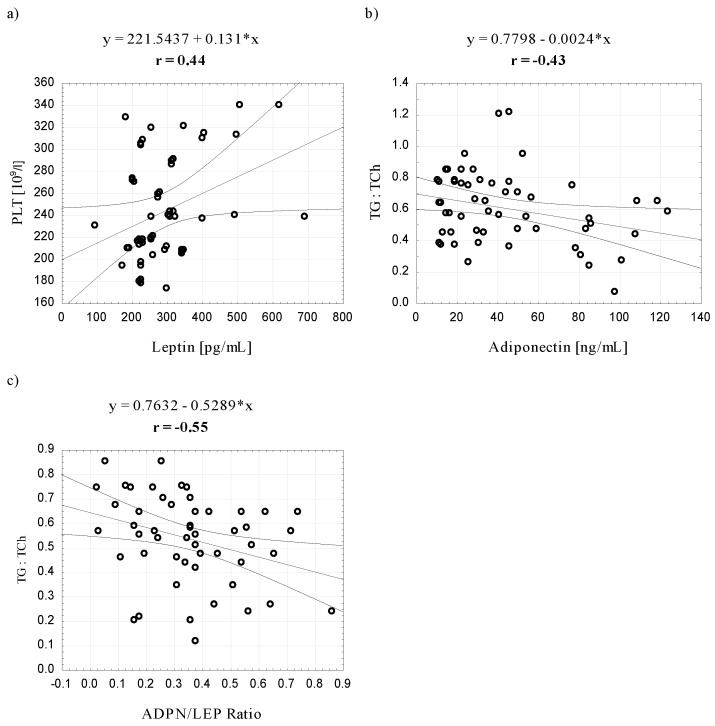
Scatter plots for the correlation (*p* < 0.05) between leptin, adiponectin and the ADPN/LEP ratio and platelet counts and the TG:TCh ratio in the group of women.

In women, we found a negative correlation between ADPN and the TG:TCh ratio only and a positive correlation between LEP and PLT. In addition, a negative correlation was observed between the ADPN/LEP ratio and the TG:TCh ratio (Figure 4). It should be noted that these relationships were not observed in men. All significant correlations demonstrated during the tests are shown in Figure 4.

## 4. Discussion

In the present study, we analyzed interactions between serum ADPN and LEP levels and fat and lean indices, hematological parameters and lipid profiles in the subjects with varying body weights. In clinical practice, the most common indicators of obesity are BMI and the waist-to-hip ratio (WHR) being measured to indicate nutritional status and used stools to correlate the risk of health problems with weight at the population level in adults. Nevertheless, these indicators, although very common and relatively easy to obtain, do not allow a precise an unambiguous definition of the relations between individual body components and adipokines. To the best of our knowledge, this is the first study that estimates the relationship between serum adiponectin and leptin concentrations and differentiates the indicators of body fat to such a detail, including a division between visceral and subcutaneous fat. Moreover, we made distinction between the trunk and lower limbs, which allows for a more complete differentiation of subcutaneous fat distribution and takes into account the division into abdominal and thigh fat (Table 1). The important assumption of the study, as planned, was to make differentiation within the body mass of participants, which was confirmed by a wide range of the BMI value, from 23.3 to 53 kg/m^3^ in all participants. The tested subjects, both women and men, varied in BMI (CVW + M = 18.4%; CVW = 19.6%; CVM = 17.8%). Importantly, despite the lack of statistically significant differences in BMI and WHR between the female group and the male one, there were significant sexual differences in terms of most adiposity parameters. Although both ADPN and LEP are mainly synthesized by adipose tissue, it has been reported that the dependence of their concentrations on the body composition does not have the same character [24]. The percentage rates of these components was highly significantly, being higher in women than in men, which is consistent with the data from literature [25]. Since we found no statistically significant dimorphism in the percentage rate of visceral adipose tissue, sexual differentiation may only concern subcutaneous fat (Table 1). Due to statistically significant higher concentrations of the analyzed adipokines in women than in men, particularly LEP (twice as high concentrations) (Table 2), subcutaneous adipose tissue may potentially regulate the concentrations of these adipokines. At the same time, however, it should be noted that both the absolute values and the percentages of lean components (LBMT, SMM) were significantly higher in men than in women.

Although numerous clinical studies show strong relationships between the described adipokines and body composition indices [26,27,28,29,30,31,32,33,34,35,36], most of them are limited to anthropometric measurements or body fat mass. Some of the studies being cited here assess body composition based only on BMI, WHR and/or the percentage of fat content, without using precise methods for detailed analysis of each component, e.g., dual-energy x-ray absorptiometry (DEXA), tomography (CT), bioelectrical impedance analysis (BIA) [25,28,33]. In our research, we took into account both fat (divided into visceral and subcutaneous adipose tissues) and lean components of the body. The obtained results highlight the high risk of error in the assessment of the relation between adipokine levels and the nutritional status and body fat when only BMI and WHR are considered and indicate the need to consider both fat and lean components.

It is known that ADPN is low in simple obesity and related disorders, such as insulin resistance, type 2 diabetes [27] and obstructive sleep apnea [17]. At the same time, high ADPN levels are observed in lean and emaciated persons, as well as in cachexia, e.g., due to cancer [37]. Some studies have shown that the level of ADPN in blood serum is inversely proportional to BMI, WHR and LEP levels in plasma [26,32,34]. The results of our study did not confirm a direct correlation between BMI and WHR and serum adipokine levels. Only the ADPN/LEP ratio negatively correlated with BMI in the entire study group, as well as independently in the group of women.

In men, the increased ADPN, LEP and ADP/LEP ratio negatively correlated with soft lean mass (SLM) and lean body mass (LBM). Interestingly, we found no relationship between adipokine levels and the indicators of adiposity in the entire study group and after division into sex groups (Table 3).

Some literature data [38] suggest that the concentration of ADPN in women can be positively correlated with subcutaneous adipose tissue located in the region of lower limbs, which could explain the relationship being obtained in our research. In order to verify this hypothesis, we evaluated the dependence of ADPN level on the absolute values and the percentages of body fat in lower limbs (BFMLR, BFMLL) in the entire group of participants and separately for women and men but we did not obtain statistically significant results (Table 3), therefore we did not confirm the results of Turer *et al.* (2011). This was perhaps due to the fact that our study group was dominated by women with android adiposity (WHR value shown in Table 1).

This study showed no significant relationship between ADPN and SFM:VFM ratio for the whole body or the trunk. According to some reports, a higher percentage rate of subcutaneous fat compared to visceral fat may be a factor for increased ADPN levels in people with obesity [25].

In our study, the LEP level strongly positively correlated with both the absolute values and the percentages of individual components of total body fat and the trunk region (BFM, VFM, SFM, BFM in the trunk and lower limbs). The obtained results lead to definite conclusions about a crucial role of adipose tissue in the release of LEP, which is further confirmed by a positive correlation between the MBF:LBM ratio and the LEP level. These results are consistent with literature data [14,26,32].

Interestingly, women in our study showed a negative correlation between LEP and the SFM:VFM ratio, which may suggest a more significant role of visceral adipose tissue than subcutaneous adipose tissue in the release of adipokines to blood.

A highly significant sexual dimorphism in LEP levels (higher concentrations in women) may be associated with higher fat indicators in women. However, it must be remembered that men and women in our study did not differ in the absolute values of fat mass indicators (Table 1 and Table 3), which further highlights the significance of relationships between subcutaneous and visceral fat and lean body mass. Although the entire study group showed a trend to abdominal obesity, which could potentially indicate a large proportion of visceral fat in body composition, the sex groups differed in the percentage rate of body fat mass in the trunk (%BFM_T_) and subcutaneous fat mass in the trunk (%SFM_T_), which may be another reason for significantly higher LEP level in women. In conclusion, it is worth noting that, despite the potential protective effect of LEP in the excess accumulation of body fat (control of the sensation of hunger and satiety), obese people have the increased levels of serum LEP; According to the present state of knowledge, this can be explained by leptin resistance [14,39].

We found no statistically significant relationship between LEP and systolic or diastolic blood pressure (BP) (Table 3) but observed a negative correlation between ADPN and systolic blood pressure (Table 3). Literature data show that ADPN and LEP seem to be possible regulators of the cardio-vascular system [19,40,41]. For example, it has been shown that when the LEP level in rats is elevated to that being observed in obesity, blood pressure and heart rate increase. The LEP administration to the cerebral ventricle or its infusion into the jugular vein of rats resulted in a dose-dependent increase in heart rate and blood pressure [42,43].

Due to the presence of LEP receptors in many tissues, e.g., in cardiomyocytes (LEPRa, -b and -c isoforms), it seems possible that LEP plays a certain role in the pathogenesis of cardiovascular diseases [16,19,39,40]. To some extent, the results obtained in animal models are supported by clinical trials. It has been shown that long-term hyperleptinemia is associated with impaired myocardial function, this condition being recognized as an independent risk factor for coronary artery disease, myocardial infarction and a predictor of subsequent cardiac events [16,44,45].

A positive correlation between LEP levels and heart rate has been reported in patients with hypertension, independent of other factors (BMI, age, insulin levels), which may suggest that increased LEP may increase BP [16,28]. However, the relationships between LEP and BP are ambiguously described in literature. Some authors have demonstrated a positive correlation between LEP and systolic and diastolic blood pressure [46], which is contrary to the data reported by Momin *et al.* whose results indicate a vasodilatory effect of LEP and, consequently, an LEP-induced reduction in BP [23]. The potential regulatory function of LEP in the cardiovascular system is still a matter of controversy and the current state of knowledge does not allow for definitive conclusions.

Literature data also suggests a potentially opposite effect of ADPN on blood pressure. The ADPN-induced decrease in BP is probably related to the renin-angiotensin system and the sympathetic nervous system [47]; in addition, the impact of ADPN on vascular tension appears to be independent of other hypertension risk factors [48]. Moreover, in animal studies and clinical trials, hypoadiponectinemia has been associated with pathological hypertrophy of the left ventricle [49].

The potential role of LEP and ADPN in the pathology of cardiovascular diseases is not limited to the impact on blood pressure. Another important issue is their effect on the metabolism and transport of lipids and, consequently, their effect on the lipid profile and the regulation of vascular endothelial inflammation. It is postulated that LEP may be involved in the body's response to stress; for example, LEP can play a role of acute phase protein, adapting the cellular metabolism to stress, e.g., in myocardial infarction [50]. This is confirmed by the elevated concentrations of LEP within the first 24 hours of infarction [43,50]. Furthermore, the LEP injection stimulates local inflammatory responses [43]. Literature data show a strong link between LEP and increased CRP independently of body weight and the occurrence of cardiovascular disease [38,51,52].

The results of our study indicate a significant correlation between serum LEP and white blood cell count, including granulocytes. Interestingly, a positive correlation was observed between serum LEP and platelet count in the entire study group and in women. It is well known that all of these factors contribute to initiation and development of atherosclerotic vessels, which confirms the previously mentioned relationship between adipokines and vascular endothelium pathology. The pathogenesis of atherosclerosis is associated with chronic inflammation within the vascular wall, endothelial dysfunction [53] and hypercoagulability state being associated with excessive platelet activation, which can explain the regularities observed in our study group.

In contrast, the anti-inflammatory activity of ADPN may protect against initiation and development of atherosclerosis and other cardiovascular pathologies. It may reduce the expression of endothelial adhesion molecules, vascular cell adhesion molecule-1 (VCAM-1), E-selectin, and intercellular adhesion molecule-1 (ICAM-1), which are receptors for monocytes, inhibiting the monocyte adhesion to the vessel wall [35,54]. Additionally, ADPN may exert a protective effect through direct inhibition of TNF-α biosynthesis by macrophages and reduction of their phagocytic properties. Moreover, it shows an inhibitory effect with respect to macrophage scavenger receptor A, therefore inhibiting the conversion of macrophages into foam cells [55]. ADPN increases nitric oxide production in the vascular endothelium and stimulates angiogenesis [56,57].

Decreased ADPN levels in obesity, type 2 diabetes and metabolic syndrome may be a cause for the development of micro-and macro-vascular diabetic and cardiovascular complications. Literature shows the advantage of the ADPN/LEP ratio over individual ADPN and LEP levels in predicting obesity complications, such as insulin resistance and endothelial damage [58] in patients without hyperglycemia [59]. However, despite all literature data, our study group demonstrated no significant correlations between ADPN and blood biochemical indices.

Analysis of the lipid profile is one of the basic diagnostic tests demonstrating the risk of atherosclerosis and metabolic syndrome. Our study group was characterized by diverse values of total cholesterol (CVW + M = 20.5%; CVW = 17.6%; CVM = 25.4%), LDL (CVW + M = 28.8%; CVW = 21.8%; CVM = 39.4%) and HDL (CVW + M = 25%; CVW = 21%; CVM = 27%), with a trend to low HDL (<40 mg/dL) and elevated LDL (>100 mg/dL) and total cholesterol (>200 mg/dL).

The ADPN and LEP levels seem to correlate only with selected parameters (TG, HDL and LDL) and in a contradictory manner. Clinical studies have demonstrated a positive correlation between ADPN and total cholesterol, HDL [25,35,60,61] and LDL [35] and a negative correlation with TG [33,34,35,60], which is confirmed by the results of our study. ADPN seems to be a strong and independent protector of cardiovascular events [62].

Despite the link between LEP and lipid metabolism and the indication that high LEP concentrations are biomarkers of simple obesity [35], our study did not demonstrate a relationship between LEP and lipid profile indices. The results presented in literature are also ambiguous and further research in this area are necessary [63,64,65].

Literature data suggest that the ADPN/LEP ratio is a sensitive risk indicator of metabolic syndrome in patients with overweight and obesity [35,66,67]. The character of our study does not allow for explicit reference to this suggestion, although some of our subjects did show strong symptoms of metabolic syndrome. With respect to the lipid profile, as an essential component of metabolic syndrome diagnosis, we observed a negative correlation between the ADPN/LEP ratio and the TG/TCH ratio in women (Figure 3). Men with high ADPN/LEP ratio exhibited lower TG and TG/HDL ratio and higher HDL compared to patients with low ADPN/LEP ratio, regardless of waist circumference [35]. The reason for inconsistent results may be the sexual dimorphism in body fat distribution mentioned above (Table 1) and the influence of hormones, e.g., inhibitory effect of androgens on ADPN and LEP levels [67], which may affect the sensitivity of the ADPN/LEP ratio indicator.

## 5. Conclusions

We are aware of the limitations of the study. The size of particular groups of women and men should be increased, allowing for age subgroups within sex and hormonal balance, particularly in premenopause, perimenopause and postmenopause in women, which could significantly affect the study results. Furthermore, the anthropometric measurements in the presented study were performed with the BIA method; it would be good to carry out a comparative analysis with the data being obtained with more objective methods of body composition analysis, for example with DEXA.

The current state of knowledge on the role of ADPN and LEP and their relationships in a variety of physiological and pathological states, though systematically supplemented by new findings, is still a subject of much controversy and confusion.

The composition of body components (not just fat components), their absolute levels and proportions, and their distribution in the human body, all seem to be crucial for blood LEP and ADPN levels. ADPN tended to depend on the absolute values of lean mass parameters, which may indicate that distribution of this adipokine depends on localized soft lean mass. In contrast, the LEP levels were obviously dependent on fat components, both their mass and proportion. Despite a common site of origin of the described adipokines, their functions and effects on body weight regulation and the pathophysiology of cardiovascular diseases are different and specific. ADPN exhibits a clearly protective action, in contrast to LEP which seems to be a predictor of many diseases. Appropriate LEP and ADPN concentrations appear to be an important part of blood pressure regulation, lipid profile and vascular endothelium inflammation, producing significant changes in the cardiovascular system. Given the current state of knowledge, the potential significance of the studied adipokines, especially ADPN and the ADPN/LEP ratio, as a valuable complementary diagnostic element in the prediction and prevention of cardiovascular diseases needs further research.

## References

[1] O’Donnell C.J., Elosua R. (2008). Cardiovascular risk factors. Insights from Framingham. Rev. Esp. Cardiol..

[2] Kershaw E.E., Flier J.S. (2004). Adipose tissue as an endocrine organ. J. Clin. Endocrinol. Metab..

[3] Giorgino F., Laviola L., Eriksson J.W. (2005). Regional differences of insulin action in adipose tissue: Insights from *in vivo* and *in vitro* studies. Acta Physiol. Scand..

[4] Yamauchi T., Waki H., Kamon J., Murakami K., Motojima K., Komeda K., Miki H., Kubota N., Terauchi Y., Tsuchida A. (2001). Inhibition of RXR and PPAR gamma ameliorates diet-induced obesity and type 2 diabetes. J. Clin. Invest..

[5] Steppan C.M., Bailey S.T., Bhat S., Brown E.J., Banerjee R.R., Wright C.M., Patel H.R., Ahima R.S., Lazar M.A. (2001). The hormone resistin links obesity to diabetes. Nature.

[6] Rajala M.W., Scherer P.E. (2003). Minireview: The adipocyte—At the crossroads of energy homeostasis, inflammation, and atherosclerosis. Endocrinology.

[7] Lyon C.J., Law R.E., Hsueh W.A. (2003). Minireview: Adiposity, inflammation, and atherogenesis. Endocrinology.

[8] Lapidus L., Bengtsson C., Larsson B., Pennert K., Rybo E., Sjöström L. (1984). Distribution of adipose tissue and risks of cardiovascular disease and death: A 12-year follow up of participants in the population study of women in Gothenburg, Sweden. Br. Med. J..

[9] Friedman J.M., Halaas J.L. (1998). Leptin and the regulation of body weight in mammals. Nature.

[10] Jaffer I., Riederer M., Shah P., Peters P., Quehenberger F., Wood A., Scharnagl H., März W., Kostner K.M., Kostner G. (2012). Expression of fat mobilizing genes in human epicardial adipose tissue. Atherosclerosis.

[11] Piñeiro R., Iglesias M.J., Gallego R., Raghay K., Eiras S., Rubio J., Diéguez C., Gualillo O., González-Juanatey J.R., Lago F. (2005). Adiponectin is synthesized and secreted by human and murine cardiomyocytes. FEBS Lett..

[12] Tomas E., Tsao T.S., Saha A.K., Murrey H.E., Zhang C.C., Itani S.I., Lodish H.F., Ruderman N.B. (2002). Enhanced muscle fat oxidation and glucose transport by ACRP30 globular domain: Acetyl-CoA carboxylase inhibition and AMP-activated protein kinase activation. Proc. Natl. Acad. Sci. USA.

[13] Yamauchi T., Kamon J., Minokoshi Y., Ito Y., Waki H., Uchida S., Yamashita S., Noda M., Kita S., Ueki K. (2002). Adiponectin stimulates glucose utilization and fatty-acid oxidation by activating AMP-activated protein kinase. Nat. Med..

[14] Shimizu H., Oh-I S., Okada S., Mori M. (2007). Leptin resistance and obesity. Endocr. J..

[15] Lam Q.L.K., Lu L. (2007). Role of leptin in immunity. Cell. Mol. Immunol..

[16] Lago F., Dieguez C., Gómez-Reino J., Gualillo O. (2007). The emerging role of adipokines as mediators of inflammation and immune responses. Cytokine Growth Factor Rev..

[17] Canavan B., Salem R.O., Schurgin S., Koutkia P., Lipinska I., Laposata M., Grinspoon S. (2005). Effects of physiological leptin administration on markers of inflammation, platelet activation, and platelet aggregation during caloric deprivation. J. Clin. Endocrinol. Metab..

[18] Frank S., Stallmeyer B., Kämpfer H., Kolb N., Pfeilschifter J. (2000). Leptin enhances wound re-epithelialization and constitutes a direct function of leptin in skin repair. J. Clin. Invest..

[19] Abe Y., Ono K., Kawamura T., Wada H., Kita T., Shimatsu A., Hasegawa K. (2007). Leptin induces elongation of cardiac myocytes and causes eccentric left ventricular dilatation with compensation. Am. J. Physiol. Heart. Circ. Physiol..

[20] Correia M.L., Morgan D.A., Sivitz W.I., Mark A.L., Haynes W.G. (2001). Leptin acts in the central nervous system to produce dose-dependent changes in arterial pressure. Hypertension.

[21] Momin A.U., Melikian N., Shah A.M., Grieve D.J., Wheatcroft S.B., John L., Gamel A.E., Desai J.B., Nelson B., Driver C. (2006). Leptin is an endothelial-independent vasodilator in humans with coronary artery disease: Evidence for tissue specificity of leptin resistance. Eur. Heart. J..

[22] Verovská R., Lacnák Z., Haluzíková D., Fábin P., Hájek P., Horák L., Haluzík M., Svacina S., Matoulek M. (2009). Comparison of various methods of body fat analysis in overweight and obese women. Vnitr. Lek..

[23] Fürstenberg A., Davenport A. (2011). Comparison of multifrequency bioelectrical impedance analysis and dual-energy X-ray absorptiometry assessments in outpatient hemodialysis patients. Am. J. Kidney. Dis..

[24] Al Mutairi S., Mojiminiyi O.A., Al Alawi A., Al Rammah T., Abdella N. (2014). Study of leptin and adiponectin as disease markers in subjects with obstructive sleep apnea. Dis. Markers.

[25] Guenther M., James R., Marks J., Zhao S., Szabo A., Kidambi S. (2014). Adiposity distribution influences circulating adiponectin levels. Transl. Res..

[26] Hsieh C.J., Wang P.W., Chen T.Y. (2014). The relationship between regional abdominal fat distribution and both insulin resistance and subclinical chronic inflammation in non-diabetic adults. Diabetol. Metab. Syndr..

[27] Silha J.V., Krsek M., Skrha J.V., Sucharda P., Nyomba B.L., Murphy L.J. (2003). Plasma resistin, adiponectin and leptin levels in lean and obese subjects: Correlations with insulin resistance. Eur. J. Endocrinol..

[28] Beltowski J. (2006). Leptin and atherosclerosis. Atherosclerosis.

[29] Horlick M.B., Rosenbaum M., Nicolson M., Levine L.S., Fedun B., Wang J., Pierson R.N., Leibel R.L. (2000). Effect of puberty on the relationship between circulating leptin and body composition. J. Clin. Endocrinol. Metab..

[30] Rosenbaum M., Nicolson M., Hirsch J., Heymsfield S.B., Gallagher D., Chu F., Leibel R.L. (1996). Effects of gender, body composition, and menopause on plasma concentrations of leptin. J. Clin. Endocrinol. Metab..

[31] Zhao Y.N., Li Q., Li Y.C. (2014). Effects of body mass index and body fat percentage on gestational complications and outcomes. J. Obstet. Gynaecol. Res..

[32] Suyila Q., Cui H., Yang L., Zhao L., Zhang R., Su X. (2013). Serum leptin concentrations in Mongolian women. Obes. Res. Clin. Pract..

[33] Kosacka M., Korzeniewska A., Jankowska R. (2013). The evaluation of body composition, adiponectin, C-reactive protein and cholesterol levels in patients with obstructive sleep apnea syndrome. Adv. Clin. Exp. Med..

[34] Goropashnaya A.V., Herron J., Sexton M., Havel P.J., Stanhope K.L., Plaetke R., Mohatt G.V., Boyer B.B. (2009). Relationships between plasma adiponectin and body fat distribution, insulin sensitivity, and plasma lipoproteins in Alaskan Yup’ik Eskimos: The Center for Alaska Native Health Research study. Metabolism.

[35] Vega G.L., Grundy S.M. (2013). Metabolic risk susceptibility in men is partially related to adiponectin/leptin ratio. J. Obes..

[36] Jürimäe T., Sudi K., Jürimäe J., Payerl D., Rüütel K. (2003). Relationships between plasma leptin levels and body composition parameters measured by different methods in postmenopausal women. Am. J. Hum. Biol..

[37] Diakowska D., Markocka-Mączka K., Szelachowski P., Grabowski K. (2014). Serum levels of resistin, adiponectin, and apelin in gastroesophageal cancer patients. Dis. Markers.

[38] Gómez-Ambrosi J., Salvador J., Páramo J.A., Orbe J., de Irala J., Diez-Caballero A., Gil M.J., Cienfuegos J.A., Frühbeck G. (2002). Involvement of leptin in the association between percentage of body fat and cardiovascular risk factors. Clin. Biochem..

[39] Karbowska J., Kochan Z. (2012). Leptin as a mediator between obesity and cardiac dysfunction. Postepy Hig. Med. Dosw..

[40] Akasaka Y., Tsunoda M., Ogata T., Ide T., Murakami K. (2010). Direct evidence for leptin-induced lipid oxidation independent of long-form leptin receptor. Biochim. Biophys. Acta..

[41] Hong S.J., Park C.G., Seo H.S., Oh D.J., Ro Y.M. (2004). Associations among plasma adiponectin, hypertension, left ventricular diastolic function and left ventricular mass index. Blood Press..

[42] Shek E.W., Brands M.W., Hall J.E. (1998). Chronic leptin infusion increases arterial pressure. Hypertension.

[43] Heymsfield S.B., Greenberg A.S., Fujioka K., Dixon R.M., Kushner R., Hunt T., Lubina J.A., Patane J., Self B., Hunt P. (1999). Recombinant leptin for weight loss in obese and lean adults: A randomized, controlled, dose-escalation trial. JAMA.

[44] Wallace A.M., McMahon A.D., Packard C.J., Kelly A., Shepherd J., Gaw A., Sattar N. (2001). Plasma leptin and the risk of cardiovascular disease in the west of Scotland coronary prevention study (WOSCOPS). Circulation.

[45] Söderberg S., Colquhoun D., Keech A., Yallop J., Barnes E.H., Pollicino C., Simes J., Tonkin A.M., Nestel P. (2009). Leptin, but not adiponectin, is a predictor of recurrent cardiovascular events in men: Results from the LIPID study. Int. J. Obes..

[46] Park K.G., Park K.S., Kim M.J., Kim H.S., Suh Y.S., Ahn J.D., Park K.K., Chang Y.C., Lee I.K. (2004). Relationship between serum adiponectin and leptin concentrations and body fat distribution. Diabetes Res. Clin. Pract. Suppl..

[47] Hall J.E. (2003). The kidney, hypertension, and obesity. Hypertension.

[48] Chow W.S., Cheung B.M.Y., Tso A.W.K., Xu A., Wat N.M.S., Fong C.H.Y., Ong L.H.Y., Tam S., Tan K.C.B., Janus E.D. (2007). Hypoadiponectinemia as a predictor for the development of hypertension: A 5-year prospective study. Hypertension.

[49] Shibata R., Ouchi N., Ito M., Kihara S., Shiojima I., Pimentel D.R., Kumada M., Sato K., Schiekofer S., Ohashi K. (2004). Adiponectin-mediated modulation of hypertrophic signals in the heart. Nat. Med..

[50] Shamsuzzaman A.S.M., Winnicki M., Wolk R., Svatikova A., Phillips B.G., Davison D.E., Berger P.B., Somers V.K. (2004). Independent association between plasma leptin and C-reactive protein in healthy humans. Circulation.

[51] Kazumi T., Kawaguchi A., Hirano T., Yoshino G. (2003). C-reactive protein in young, apparently healthy men: Associations with serum leptin, QTc interval, and high-density lipoprotein-cholesterol. Metabolism.

[52] Haffner S.M., Mykkänen L., Festa A., Burke J.P., Stern M.P. (2000). Insulin-resistant prediabetic subjects have more atherogenic risk factors than insulin-sensitive prediabetic subjects: Implications for preventing coronary heart disease during the prediabetic state. Circulation.

[53] Ouchi N., Kihara S., Arita Y., Maeda K., Kuriyama H., Okamoto Y., Hotta K., Nishida M., Takahashi M., Nakamura T. (1999). Novel modulator for endothelial adhesion molecules: Adipocyte-derived plasma protein adiponectin. Circulation.

[54] Ouchi N., Kihara S., Arita Y., Nishida M., Matsuyama A., Okamoto Y., Ishigami M., Kuriyama H., Kishida K., Nishizawa H. (2001). Adipocyte-derived plasma protein, adiponectin, suppresses lipid accumulation and class A scavenger receptor expression in human monocyte-derived macrophages. Circulation.

[55] Chandran M., Phillips S.A., Ciaraldi T., Henry R.R. (2003). Adiponectin: More than just another fat cell hormone?. Diabetes Care.

[56] Díez J.J., Iglesias P. (2003). The role of the novel adipocyte-derived hormone adiponectin in human disease. Eur. J. Endocrinol..

[57] Kieć-Klimczak M., Malczewska-Malec M., Huszno B. (2008). Leptin to adiponectin ratio, as an index of insulin resistance and atherosclerosis development. Przegl. Lek..

[58] Inoue M., Yano M., Yamakado M., Maehata E., Suzuki S. (2006). Relationship between the adiponectin-leptin ratio and parameters of insulin resistance in subjects without hyperglycemia. Metabolism.

[59] Baratta R., Amato S., Degano C., Farina M.G., Patanè G., Vigneri R., Frittitta L. (2004). Adiponectin relationship with lipid metabolism is independent of body fat mass: Evidence from both cross-sectional and intervention studies. J. Clin. Endocrinol. Metab..

[60] El-Shafey E.M., Shalan M. (2014). Plasma adiponectin levels for prediction of cardiovascular risk among hemodialysis patients. Ther. Apher. Dial..

[61] Zoccali C., Mallamaci F., Tripepi G., Benedetto F.A., Cutrupi S., Parlongo S., Malatino L.S., Bonanno G., Seminara G., Rapisarda F. (2002). Adiponectin, metabolic risk factors, and cardiovascular events among patients with end—Stage renal disease. J. Am. Soc. Nephrol..

[62] Gray B., Steyn F., Davies P.S., Vitetta L. (2014). Liver function parameters, cholesterol, and phospholipid α-linoleic acid are associated with adipokine levels in overweight and obese adults. Nutr. Res..

[63] Mirrakhimov E., Kerimkulova A., Lunegova O., Mirrakhimov A., Alibaeva N., Nabiev M. (2014). Lipids and leptin level in natives of Kyrgyzstan. Turk. Kardiyol. Dern. Ars..

[64] Wannamethee S.G., Tchernova J., Whincup P., Lowe G.D., Kelly A., Rumley A., Wallace A.M., Sattar N. (2007). Plasma leptin: Associations with metabolic, inflammatory and haemostatic risk factors for cardiovascular disease. Atherosclerosis.

[65] Mirza S., Qu H.Q., Li Q., Martinez P.J., Rentfro A.R., McCormick J.B., Fisher-Hoch S.P. (2011). Adiponectin/leptin ratio and metabolic syndrome in a Mexican American population. Clin. Invest. Med..

[66] Yoon J.H., Park J.K., Oh S.S., Lee K.H., Kim S.K., Cho I.J., Kim J.K., Kang H.T., Ahn S.G., Lee J.W. (2011). The ratio of serum leptin to adiponectin provides adjunctive information to the risk of metabolic syndrome beyond the homeostasis model assessment insulin resistance: The Korean Genomic Rural Cohort Study. Clin. Chim. Acta.

[67] Gannagé-Yared M.H., Khalife S., Semaan M., Fares F., Jambart S., Halaby G. (2006). Serum adiponectin and leptin levels in relation to the metabolic syndrome, androgenic profile and somatotropic axis in healthy non-diabetic elderly men. Eur. J. Endocrinol..

